# Implementation and Evaluation of a Virtual Reality Simulation: Intravenous Injection Training System

**DOI:** 10.3390/ijerph19095439

**Published:** 2022-04-29

**Authors:** Ji Sun Lee

**Affiliations:** Department of Nursing, Honam University, Gwangju 62399, Korea; 2018091@honam.ac.kr

**Keywords:** virtual reality, computer simulations, education, administration intravenous

## Abstract

In nursing education, virtual reality simulation (VRS) is recognized as an effective learning method as it overcomes limitations in practical training and positively influences learning ability and satisfaction levels. The purpose of this study was to develop VRS for intravenous (IV) injection and investigate how it affects nursing students’ academic knowledge, performance confidence, and clinical practice competencies. A quasi-experimental control group pretest and post-test design was used. Participants were nursing students who either received a training system for an IV injection through VRS (experimental group; *n* = 20) or who received an IV arm simulator (control group; *n* = 20). The results revealed significantly higher knowledge (U = 156.5, *p* = 0.024) and clinical performance competency (U = 87.5, *p* = 0.002) with the procedure of using a training system of VRS for IV injection compared to having training via an IV arm simulator. This study verified that VRS for IV injection was more effective than an IV arm simulator for practical training on IV injection. Thus, VRS for IV injection, an effective teaching method used to improve learning ability and satisfaction levels, can be used as a training method in the future.

## 1. Introduction

The goal of nursing education is associated with the level of a nurse’s competency; it is necessary to connect education to real practice. The Korean Accreditation Board of Nursing Education (KABON) emphasized the necessity of proactive activities to promote qualitative improvement in nursing education and support and manage nursing students in terms of improving their performance [1]. Due to the ongoing COVID-19 crisis and widespread awareness of patient safety culture, observation-based training is being conducted instead of direct practice. As the expectations of obtaining high-quality nursing services are higher for patients who receive health care services, new nurses are also required to have excellent clinical competency [2]. Simulation training is beneficial as it can generate situations similar to those observed in practical clinical settings, thus allowing nursing skills to be practiced without harming any patients [3]. However, simulation training using mannequins requires a separate space, only a small number of students can be accommodated at a time, and it involves equipment installation and management that is expensive. Therefore, it is necessary to develop a virtual reality simulation (VRS) as a teaching method to address the abovementioned problems [4]. Virtual reality (VR) is a video technology that provides users with an environment that is similar to reality, and VRS training is referred to the method of teaching wherein students make decisions and practice their nursing skills in virtual-reality spaces based on actual nursing cases. The VRS training has the advantages of no space constraints, costs less than existing simulation training, accommodates a large number of students at the same time, and allows repetitive learning [5]. Moreover, in addition to the students’ learning outcomes and satisfaction, it positively influences knowledge, self-efficacy, communication skills, and clinical practice competencies through self-directed learning [6].

The intravenous (IV) injection is the most common medical practice conducted during drug injection and blood collection. Particularly, nurses bear a significant responsibility of IV catheter insertion, catheter management, and drugs injection; therefore, efforts should be made to train nurses to ensure minimal discomfort and improve safety for patients. The IV arm simulator commonly used to train IV injection in nursing education provides the advantages of visualizing the veins and inserting the needle directly into the vein represented by a rubber tube, but this does not completely reflect the characteristics of real veins. Furthermore, repeated practical training with this simplified model fails to result in a successful performance in practical clinical settings, causing decreased confidence among new nurses [7]. To tackle these disadvantages, IV injection training using VR was implemented, which resulted in a positive effect on knowledge, performance confidence, and performance capabilities [8,9]. However, pre-existing studies mostly focus on the haptic IV simulator and IV catheter insertion skills, leading to constraints in promoting an integrated understanding of the IV injection process, including selection and injection of drugs and calculation of infusion rates. Moreover, the problem-solving ability of the students was reported to be the biggest factor affecting clinical practice competencies [10]. Therefore, VRS is required to learn the IV injection process to allow for certain scenarios. This study aimed to develop a VRS for IV injection training system, evaluate its effectiveness among nursing students, and utilize the results as base data to establish effective teaching methods for improving competence among nursing students. The research hypotheses are as follows: First, nursing students who participated in VRS for IV injection will have increased knowledge compared with those in the control group. Second, nursing students who participated in VRS for IV injection will have an improved performance confidence compared with those in the control group. Third, nursing students who participated in VRS for IV injection will have improved clinical practice competencies compared with those in the control group.

## 2. Materials and Methods

### 2.1. Research Design

A quasi-experimental research using a nonequivalent control group pretest and post-test design was conducted to develop the VRS for IV injection and to prove its effectiveness. Participants were nursing students who either received a training system for the IV injection through VRS (experimental group) or who received IV arm simulator (control group).

### 2.2. Study Participants

The participants included senior nursing students from a nursing college in South Korea recruited through convenient sampling. The selection criteria were (1) taking practicum for nursing practice competencies in the first semester of the senior year; (2) experienced practical training for IV injection skills through a practicum for fundamental nursing in the second semester of the school year, 2019; and (3) understanding the purpose of the study and participating voluntarily. Those who had experienced an IV injection training system, which is to be applied in the study as an intervention, were excluded from the study.

The minimum sample size required for conducting the study was calculated using G*Power 3.1 Program, and the number of participants from a t-test of both groups was calculated using two-sided test, a significance level of α = 0.05, an effect size of 0.95, and a power of 0.80, requiring a minimum of 38 students. The effect size was selected based on the result value of 0.95 indicating a minimal effect from effect sizes between 0.95 and 8.8 from a previous study that investigated the effect of practical training for nursing students using virtual learning simulations. The final participants included 40 students who would take the applicable practicum and wanted to participate, considering dropouts. The participants belonged to two classes of nursing practice competencies and were classified into experimental and control groups to prevent experimental dispersion. There were no dropouts, and data from the experimental and control groups of 20 participants were used for the final analysis.

### 2.3. Research Instruments

#### 2.3.1. Knowledge

Knowledge was measured using a tool that the authors developed by modifying and complementing the items on IV injection of fundamental nursing skills as suggested by KABON [1]. The expert group comprising two nursing professors and a clinical specialist selected items only scoring ≥0.8 points of content validity index (CVI) through item validation. Knowledge comprises a total of five items, including three regarding performing venipuncture, one regarding the purpose of venipuncture, and one regarding basic knowledge of IV injection. The scores were measured as 1 point for the correct answer and 0 points for the incorrect answer; the total score ranged from 0 to 5 points. Higher scores indicate a higher level of knowledge about IV injection.

#### 2.3.2. Performance Confidence

An instrument developed by Ayres [11] and adapted by Park and Kweon [12] was approved to measure performance confidence in this study. The instrument was a 7-point scale with a total of 10 points, and the measured scores ranged from 10 to 70 points, wherein higher scores indicated higher levels of performance confidence. Regarding the reliability of the instrument, Cronbach’s α at the time of its development was 0.94, 0.95 in Park and Kweon’s study [12], and 0.93 in this study.

#### 2.3.3. Clinical Practice Competency

Clinical practice competency refers to a score measured using a fundamental nursing skill checklist for IV injection as suggested by KABON [1]. The checklist comprises a total of 30 items, scoring 2 points for “good”, 1 point for “insufficient”, and 0 points for “didn’t do it”, and the scores range from 0 to 60 points; the total score was converted into a score out of 100, and a higher score indicated a higher level of clinical competency.

### 2.4. VRS for IV Injection Training System

VRS developed by NEWBASE Inc. was used for this research. When this program was developed, researchers participated as advisors; according to Intervention Mapping Protocol (IMP), the program was developed on the conceptual basis of a simulation model for the design, development, and implementation of the simulation [13].

Step 1: Needs assessment

In the needs assessment stage, the literature was reviewed, and interviews were conducted to identify the educational needs of nursing students. The importance of evaluation skills and managing IV injection training, which is frequently performed as a basic nursing skill in clinical settings, has been verified. Among IV injection, students have demanded training on IV catheter insertion, catheter management, and drugs injection. Particularly, based on KABON’s [1] operation system and the goals of practical training, educational content, and evaluation methods, this study intended to improve the quality of practical training.

Step 2: Goal setting

**Figure 1:**
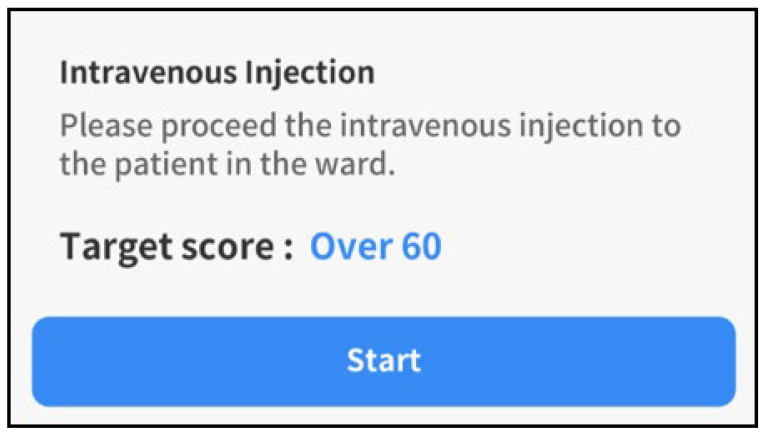
Target score in the VRS for IV injection.

For program outcomes (PO) and performance methods of VRS for IV injection, 2 of the 12 POs suggested in KABON [1], i.e., “application of integrated nursing skills” and “critical thinking development,” were selected, and the goal of education was to achieve relevant competencies. In this program, the target score was set at 60 points for all cases so that all learners could achieve a PO of intermediate or higher levels (Figure 1).

Step 3: Selecting interventional methods and strategies

An interventional method was selected to achieve behavioral changes among the participants. The participants were asked to carry electronic devices, such as smartphones, tablets, or computers to access the program.

Step 4: Program content development

**Figure 2:**
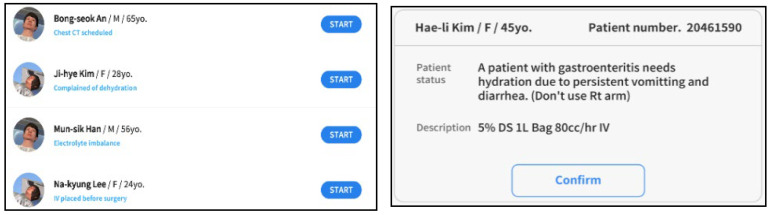
Scenarios in the VRS for IV injection.

Based on the commonly experienced cases in a clinical setting, 20 scenarios were developed, including test preparation, fluid imbalance, hyperthermia, fasting, and hypovolemic shock; among these, 10 scenarios were selected by taking into account of the needs and competency levels of the participants (Figure 2). The contents of the program included achievement goals suggested in KABON as follows: (a) the purpose of IV injection and the procedure to be followed; (b) preparation of the equipment and other items required for IV injection; (c) performing IV catheter insertion, fluid connection, and fluid injection; and (d) controlling infusion doses and rates. An expert group, comprising three clinical specialists and two nursing professors with experience in running clinical simulations in practicum and developing scenarios, modified and complemented the program through item validation and selected cases of only CVI of ≥0.8.

Step 5: Program logistics

**Figure 3:**
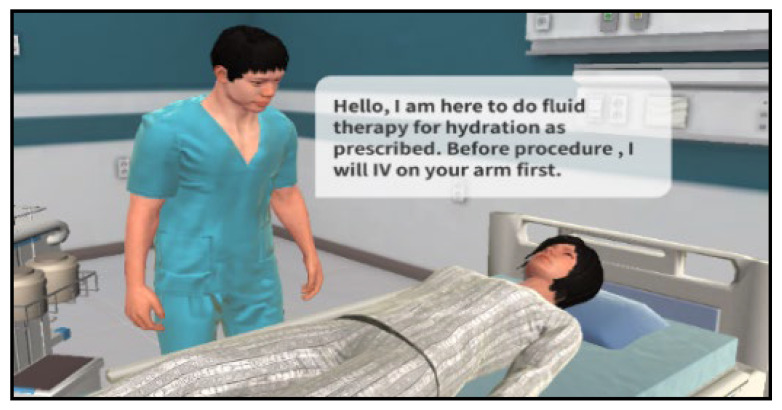
Application of scenario through VRS for IV injection.

**Figure 4:**
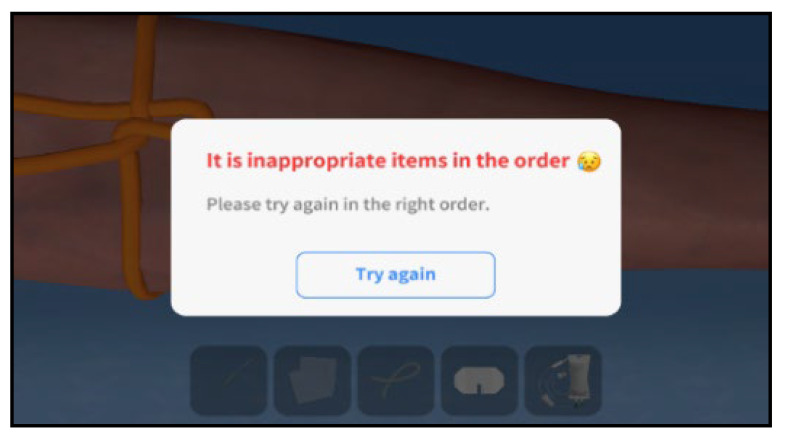
Attention alarm during VRS for IV injection.

**Figure 5:**
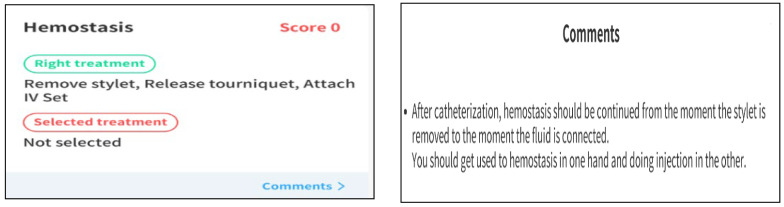
Evaluation system in the VRS for IV injection.

The practical training was initiated on the Kolb learning cycle, wherein the students were to form new cognitive structures (abstract concept) about the patient status and application of nursing skills through VRS (Figure 3) [14]. In this program, an alarm was played to call the students’ attention when a question of judgment and decision appeared in each circumstance in the given case. Additionally, at the end of learning in each case, students could check, through an evaluation system, the results of the process in which problems occurred and the problem-solving process (Figure 4). Debriefings were organized through an evaluation system structured in steps of situation description, analysis, and application. Students could check for the patient status and doctor prescriptions and utilize their nursing skills through patient cases suggested in the VRS program. Practical training was evaluated based on Kim’s study with an experimental group of 20 students classified into five teams of four students, each for 80 min at each time [15]. During the entire practical training, team members could share their experiences freely and discuss the procedures they performed well and those they did not. For evaluation and feedback after the program, each participant’s individual nursing competency and program operation were evaluated (Figure 5).

### 2.5. Data Collection and Ethical Consideration

This study was conducted according to the guidelines of the Declaration of Helsinki, and approved by the Institutional Review Board. The study was conducted from May to June, 2021. After providing a summary and explaining the purpose of the study, 40 students were selected, with their consent, as the final participants. The researchers informed the participants that the survey results would be used with utmost confidentiality and that nonparticipation would have no disadvantages; they could also refuse to participate at their own free will at any time. Informed consent and data were obtained by research assistants to protect the confidentiality of participants.

#### 2.5.1. Preliminary Survey

In May 2021, the preliminary survey of 40 participants classified into the experimental and control groups was conducted using a self-reported questionnaire about their knowledge and performance confidence. The purpose, study contents, procedures, and precautions of IV-injecting skills were explained for 20 min using a PowerPoint presentation prepared by the researchers, and the participants watched a 10 min long video that was produced according to the evaluation process in KABON [1]. Then, clinical practice competency was evaluated for each participant, using the IV training arm simulator for 10 min.

#### 2.5.2. Experimental Treatment

To prevent experimental dispersion, the experiments schedules were different for the experimental and control groups. Both groups had a total of five teams, with four people in each team; experiments were conducted in the training room to analyze their practical skills according to the schedule given to each team. First, the researcher asked the experimental group to use devices such as smartphones, tablets, or computers; demonstrated the procedure for using a VRS training system; and discussed the precautions for 20 min. Second, the experimental group attended an 80 min long class using VRS for IV injection; the class involved learning for 40 min where the instructor stayed in the training room to answer the participants’ questions, followed by self-practice for 40 min. The control group underwent training using the IV training arm simulator, with the instructor demonstrating how to use the simulator, followed by a discussion of the precautions for 20 min. The control group also underwent an 80 min class, which included learning with an instructor followed by self-practice for 40 min each.

#### 2.5.3. Post-Training Survey

Post-training surveys were conducted 3 days after interventions for each team depending on the individual schedule. Knowledge and performance confidence were surveyed using self-report questionnaires, and clinical practice competencies were evaluated in the method similar to that used in the preliminary survey. The evaluation was performed by one research assistant who had used the same evaluation tool but was blinded to the group allocation of students. To determine clinical practice competency, examiners evaluated whether the participants completed all items on the checklist and closely observed the participants while checking the checklist. After the experiments were completed, learning opportunities were provided to those who did not receive interventional treatments to ensure equal opportunities of practical training for the experimental and control groups.

### 2.6. Data Analyses

The collected data were analyzed using SPSS/WIN 25.0. According to the Kolmogorov–Smirnov and Shapiro–Wilk tests of normality, data distribution was not normal; therefore, the data were analyzed using nonparametric statistics. The test for homogeneity on participant characteristics used descriptive statistics, χ^2^-test, and Mann–Whitney U test. The differences in the average knowledge, performance confidence, and clinical practice competencies before and after the intervention were analyzed for the experimental and control groups using Wilcoxon signed-rank and Mann–Whitney U tests, respectively.

## 3. Results

### 3.1. General Characteristics

The general characteristics of the participants are presented in Table 1. The medians of the participants were 22.50 and 22.50 years in the experimental and control groups. In terms of gender, women predominated in both the experimental and control groups with 16 (80.0%) and 15 (75.0%) women, respectively. No significant difference was observed in general characteristics, indicating that they are homogeneous.

### 3.2. Effectiveness of VRS for IV Injection

**Hypothesis** **1** **(H1).** *The results for the test of the hypothesis “Nursing students who participated in VRS for IV injection will have increased knowledge compared with those in the control group” is presented in Table 2. The medians for knowledge in the experimental group were 4.00 and 5.00 points before and after intervention, respectively, and the medians in the control group were 4.50 and 5.00 points before and after intervention, respectively. According to the analysis, hypothesis 1 was supported as a significant difference was observed between the two groups (p = 0.024).*

**Hypothesis** **2** **(H2).** *The result for the test of the hypothesis “Nursing students who participated in VRS for IV injection will have an improved performance confidence compared with those in the control group” is presented in Table 2. The medians for performance confidence in the experimental group were 9.10 and 9.40 points before and after intervention, respectively, and the scores in the control groups were 9.40 and 9.40 before and after intervention, respectively. According to the analysis, hypothesis 2 was dismissed since no significant difference was observed between the two groups (p = 0.231).*

**Hypothesis** **3** **(H3).** *The result for the test of the hypothesis “Nursing students who participated in VRS for IV injection will have an improved clinical practice competencies compared to those in the control group” is presented in Table 2. The medians for clinical practice competency in the experimental group were 83.00 and 93.00 points before and after intervention, respectively, and the medians in the control groups were 84.50 and 88.00 points before and after intervention, respectively. According to the analysis, hypothesis 3 was supported as a significant difference was observed between the two groups (p = 0.002).*

## 4. Discussion

This study was conducted in an attempt to overcome the limitations of conventional face-to-face classes due to COVID-19, to provide a highly realistic educational environment, and verify the effectiveness of the training system using VRS for IV injection. VRS, which was developed based on various clinical cases, was effective for improving knowledge and clinical practice competencies among nursing students.

The study is significant in the sense that it employed VRS for an IV injection training system, and it was applied as a substitution method to improve the nursing competency of nursing students by implementing a VR training system, wherein the environment was similar to that in a clinical setting. Most practicum training for IV catheter injection using VR involves using the VR-haptic IV simulator (VR simulator), and most studies compare the differences among the training groups using a VR simulator and IV arm simulator [16]. Training with the IV arm simulator was reported to be more effective as it allowed the students to learn skills, such as IV catheter insertion [8]. Conversely, some studies reported that the VR simulator was more effective for improving clinical practice competencies than the IV arm simulator [8,16]. A study that was conducted to address the limitation of such previous studies applied a combination of the two types of simulators and determined that the group using the combination of the two simulators had higher levels of clinical practice competencies than those who used each simulator individually [16]. This is a result that supports the research findings that blended learning improves the outcomes of nursing education [17].

VR training is a teaching method that uses VRS based on clinical case scenarios that train students to make decisions and provides different scenarios for performing nursing skills [18]. However, the training system used by most studies focused on gaining nursing skills. To complement the limitations of the previous studies and not only gain nursing skills but also improve practical capabilities in terms of IV injection, this study developed a VRS for IV injection. This VRS allowed us to conduct the same training courses as an actual simulation learning course simply by accessing a website and without the use of expensive equipment.

The experimental group which underwent the VRS showed significantly higher levels of knowledge than those in the control group using the IV arm simulator. Although this is similar to a previous study reporting that VR simulator learning significantly affects knowledge [8], another study reported that no significant difference was observed [18]. Accordingly, it can be assumed that the differences in the results of previous studies were caused by various tools used to measure knowledge and the different study designs, such as participants and interventional methods. Conversely, VRS learning motivation in students, organizes knowledge in a clinical context, promotes self-learning, and increases cognitive power, including problem-solving skills. A VRS can be considered useful for students as it encourages cognitive learning through repeated self-driven learning-based on scenario-based learning, leading to improved knowledge levels. A repeated simulation experience can be an effective strategy to increase knowledge since it results in the expansion of a concept by adding new experience to it [17]. In particular, considering that knowledge in nursing is an essential factor for nurses to practice and make a clinical judgment, applying VRS in nursing practice will improve nursing competencies.

No significant differences were observed in performance confidence between the experimental and control groups. This was equivalent to previous studies reporting that IV catheter insertion using a VR simulator was not effective for increasing performance confidence [19]. Some studies support these results and reported that the learning process with a VR simulator allows for a repeated and independent learning and increases confidence by safely conducting the process [20]. Unlike some previous studies, that study showed no significant difference in terms of performance confidence. It was found that the training groups using VRS and the IV arm simulator were compared in this study, but the study showing an increased performance confidence used combined, practical training [21]. In other words, by using combined practical training, students experienced practical skills through an IV arm simulator, and various experiences with clinical cases through VRS improved the students’ thinking skills, leading to stability and improved performance confidence [22].

A number of studies have already reported that a VR simulator facilitates repeated practice that enhances competency without spending instructional hours [21]. Through VR simulator training, students not only required fewer attempts to successfully complete IV catheter insertion but also required less total time [20,22]. IV catheter insertion is performed to allow drug administration and teaches a standardized method that includes practicing IV injection. KABON suggested the necessity of practicing nursing skills as achievement goals for IV injection, in which nursing competencies are reflected, including IV catheter insertion and its connection, practice of fluid injection, and control of IV fluid infusion dosage and rates [1]. In this study, clinical practice competencies were measured using an evaluation tool that reflected these achievement goals, revealing that the experimental group had significantly higher levels of clinical practice competencies than the control group. Although our study cannot be compared to that by Kim and Kim [8], which evaluated practical skills for IV catheter insertion, it is notable that our study, which applied only VRS and evaluated clinical practice competencies for IV injection, showed that clinical practice competencies in the experimental group were high, with a score of 93.0 points (out of 100); this is a higher score compared to the 55.32 points (out of 100) in the study by Kim and Kim [8]. Such differences are surely influenced by the participants and interventional methods; however, this can be used as empirical evidence that VRS can only have educational effectiveness.

The experimental group, which underwent IV injection via a VRS, showed significantly higher levels of clinical practice competencies than the control group. These results were similar to those of a study reporting that the VR simulator training group received the highest scores in performing venipuncture compared to those in the IV arm simulator training group [19]. Another study examining clinical practice competencies using the same tools investigated the practical IV catheter insertion skills of a combined training group using a VR simulator and an IV arm simulator and one using an arm simulator alone [9]. The results showed that the combined training group had higher levels of clinical practice competencies.

Previous studies use a VR simulator to train for an IV injection based on the haptic system while watching a computer screen [9,19]. There are several advantages to this system, including an interface where students can directly palpate the vein, stretch the skin, and feel the resistance during venipuncture; however, the disadvantages of this system are that it cannot simulate real touch similar to a person’s veins when inserting an IV catheter and requires expensive equipment [18]. The VRS employed in our study allows an intuitive and lively learning from the first-person point of view in VR, which is similar to that in actual clinical settings, without requiring any equipment preparation. Furthermore, another advantage is that learners can train themselves without any constraints of time and space and based on various patient cases. However, as all the procedures are performed using icons and function keys displayed in the program, it can feel out of touch with reality, and applying it to the real world can be difficult.

However, in VR, potential digital bits are realized as analog images through perception, and the presence inside the virtual world allows for an adaption to the real world. Accordingly, it is expected that knowledge will increase, and nursing competencies will expand, if a learning program that combines virtual variables with an actual existing space in reality is created [23]. In addition, VRS can be used as an effective learning method because it overcomes the limitations of practical education and has a positive effect on learning ability and satisfaction [24].

This study employed VRS for students who had experienced practical training for IV injection through a practicum for fundamental nursing to enhance their practical competencies before graduation, thereby showing great educational effectiveness even though an IV arm simulator was not used in combination. In the future, studies considering students’ experience, competencies for practical skills, and learning environments are required. A systematic literature review on IV injection skills reported that the combination of a VR simulator and an IV arm simulator was the most effective [25], and utilizing multiple methods according to the purposes of a study and education is necessary.

This study has several limitations. First, the results cannot be generalized because the students from one college were recruited using convenience sampling according to the operation of the curriculum. Second, students from one college were included in the experimental and control groups to control exogenous variables that may occur due to differences in the curriculum that may arise when students from another college are used as a control group, the dispersion effects cannot be completely excluded. Third, as the PO may differ based on the individual participant, the interpretation of results should be prudent, and further studies are needed. Finally, this study used VRS for students who had practically experienced IV injection. Therefore, the effects may differ when applied to students who did not experience the relevant skills, and caution should be used in applying these results.

## 5. Conclusions

Based on the findings of this study, the following suggestions are made: First, repeated studies on a large sample size are suggested to determine the effects of VRS training system for IV injection. Second, studies must be conducted to evaluate the effects of a program using a combination of VR and conventional education methods. Third, investigating outcomes including various fundamental nursing competencies is suggested to evaluate the effectiveness of a program. Fourth, conducting a qualitative study is suggested to explore the learning experiences of nursing students who underwent VRS for IV injection.

## Figures and Tables

**Table 1:** General characteristics of participants (*n* = 40).

Characteristics	Categories	Exp. (*n* = 20)	Cont. (*n* = 20)	U or *χ*^2^	*p*-Value
		Median (IQR)/*n* (%)			
Age(years)		22.50 (1.00)	22.50 (2.00)	208.50	0.820 ^‡^
Gender	Male	4 (20)	5 (25.0)	1.40	0.705 ^†^
	Female	16 (80.0)	15 (75.0)		
GPA	3.0~3.4	1 (5.0)	3 (15.0)	1.14	0.709 ^†^
	3.5~3.9	14 (70.0)	12 (60.0)		
	≥4.0	5 (25.0)	5 (25.0)		
Satisfaction with one’s major	Satisfaction	8 (40.0)	7 (35.0)	0.24	0.708 ^†^
	Common	9 (45.0)	10 (50.0)		
	Unsatisfaction	3 (15.0)	3 (15.0)		
Satisfaction with practical training	Satisfaction	11 (55.0)	9 (45.0)	0.70	0.805 ^†^
	Common	8 (40.0)	9 (45.0)		
	Unsatisfaction	1 (5.0)	2 (10.0)		
Knowledge		4.00 (1.75)	4.50 (1.00)	243.0	0.253 ^‡^

Exp. = experimental group; Cont. = control group; IQR = interquartile range; GPA = grade point average. ^‡^ Mann–Whitney U test; ^†^ Fisher’s exact test.

**Table 2:** Comparison of knowledge, performance confidence and clinical practice competencies within two groups (*n* = 40).

Characteristics	Groups	Pre-Test	Post-Test	*Z* (*p*-Value)	Difference	*U* (*p*-Value)
		Median (IQR)			Median (IQR)	
Knowledge	Exp.	4.00 (1.75)	5.00 (0.75)	3.15 (0.002) **	0.50 (1.00)	156.5 (0.024) *
	Cont.	4.50 (1.00)	5.00 (1.00)	2.76 (0.006) **	0.00 (0.00)	
Performance confidence	Exp.	9.10 (2.15)	9.40 (0.75)	2.42 (0.015) *	0.10 (1.35)	155.0 (0.231)
	Cont.	9.40 (1.75)	9.40 (1.18)	0.59 (0.553)	0.15 (0.00)	
Clinical practice competencies	Exp.	83.00 (9.50)	93.00 (7.00)	3.94 (0.015) *	7.00 (5.50)	87.5 (0.002) **
	Cont.	84.50 (10.00)	88.00 (13.00)	3.97 (<0.001) ***	4.00 (2.50)	

Exp. = experimental group; Cont. = control group; IQR = interquartile range; GPA = grade point average. * *p* < 0.05; ** *p* < 0.01; *** *p* < 0.001.

## Data Availability

The data presented in this study are available on request from the corresponding author. The data are not publicly available due to restrictions, e.g., privacy or ethical.
